# Associations between objectively assessed and questionnaire-based sedentary behaviour with body mass index and systolic blood pressure in Kuwaiti adolescents

**DOI:** 10.1186/s13104-019-4626-0

**Published:** 2019-09-18

**Authors:** Rawan Hashem, Juan Pablo Rey-Lόpez, Mark Hamer, Anne McMunn, Alex Rowlands, Peter H. Whincup, Christopher G. Owen, Ding Ding, Lauren Powell, Emmanuel Stamatakis

**Affiliations:** 1 Department of Epidemiology and Public Health, University College London, London, WC1E 6BT UK; 2 Charles Perkins Centre, Prevention Research Collaboration, School of Public Health, University of Sydney, Sydney, NSW Australia; 3 School of Sport Exercise & Health Sciences, Loughborough University, Loughborough, LE11 3TU UK; 4 Diabetes Research Centre, University of Leicester, Leicester, UK; 5 NIHR Leicester-Loughborough Diet, Lifestyle and Physical Activity Biomedical Research Unit, Leicester, UK; 6 Alliance for Research in Exercise, Nutrition and Activity (ARENA), Sansom Institute for Health Research, Division of Health Sciences, University of South Australia, Adelaide, Australia; 7 Population Health Research Institute, St George’s University of London, London, UK

**Keywords:** Blood pressure, Body mass index, Adiposity, Accelerometry, Sedentary behavior

## Abstract

**Objective:**

Kuwait has one of the highest obesity rates in the world. This study examined the associations between sedentary behaviour (objectively measured and self-reported), adiposity and systolic blood pressure in a sample of adolescents residing in Kuwait. Data was obtained from the Study of Health and Activity among adolescents in Kuwait (2012–2013). The sample included a total of 435 adolescents (201 boys). Outcomes were age- and sex specific body mass index Z-scores and systolic blood pressure. Exposures were total sedentary behaviour measured by accelerometry and time spent in some sedentary behaviours (television viewing, video games, computer use and total screen-time). We used multiple linear regression analyses, adjusted for age, governorate, maternal education and physical activity, to examine associations between sedentary behaviour and adiposity and systolic blood pressure.

**Results:**

Only 2 statistically significant associations were found between sedentary behaviour and the study outcomes: body mass in boys was directly associated with higher sedentary time [β (95% CIs) 0.003 (0.00 to 0.06)]; body mass index was inversely associated with videogames in both sexes [girls: β (95% CIs) − 0.17 (− 0.48 to − 0.04); boys: − 0.24 (− 0.57 to − 0.12)]. In this sample of Kuwaiti adolescents, sedentary behaviour showed limited deleterious associations with adiposity and systolic blood pressure.

## Introduction

The epidemic of overweight and obesity affects almost all nations and age groups. In 2013, 36.9% of men and 38.0% of women had a body mass index (BMI) ≥ 25 [1]. In Kuwait, the prevalence of overweight and obesity in adults, [1] and adolescents, [2] has increased dramatically over the last two decades: 74% of Kuwaiti men (> 20 years), 84% of women, 55% of boys and 49% of girls are considered overweight or obese. To reduce obesity in Kuwait, it is important to identify the major factors contributing to the obesity epidemic.

Sedentary behaviour (SB, characterised by very low energy expenditure in a reclined or seated position) [3] has been proposed as an independent risk factor of overweight and impaired cardio-metabolic health [4]. However, in children and adolescents, the observational evidence supporting this assertion is limited and equivocal. For example, several recent reviews have concluded that objectively measured sedentary time in children and adolescents is unrelated to adiposity markers (e.g., BMI), [5, 6] or blood pressure [6]. Yet, some SB types such as television (TV) viewing have been associated with adiposity, [5, 7, 8] even after adjustment for moderate to vigorous physical activity (MVPA). Time spent using the internet, [9] or playing with videogames, [8, 10] has also been positively associated with adiposity, yet for videogames the literature is inconsistent [11, 12]. The current SB literature is limited by a reliance on questionnaires to assess SB time. Most studies have also been conducted in high-income countries. Studies of SB in different geographical areas that include both objective and questionnaire-based measurements may provide a better understanding of the role of SB in adolescent’s health.

Using a sample of adolescents living in Kuwait, the aim of this study was to examine the association between objectively measured and questionnaire-based SB and adiposity levels and systolic blood pressure.

## Main text

### Materials and methods

#### Study design

The Study of Health and Activity among Adolescents in Kuwait (SHAAK) [13] is a cross-sectional study of Kuwait adolescents. The main objective of SHAAK study was to describe the prevalence of obesity, physical inactivity and time spent in SB in adolescents, and to examine their associations with cardiovascular risk factors.

#### Participants

The study population was drawn from several governorates of Kuwait (Hawalli, Asimah, Jahra, Farwaniya, Ahmadi and Mubarak), and included adolescents of both sexes and several school grades (7 to 12). Researchers randomly selected schools for each sex and governorate using a serial number assigned to each school. Using a similar allocation process, one class was selected from each grade (three classes from each intermediate and secondary school). Data collection occurred between October 2012 and June 2013, following a standard protocol. 594 students were initially invited to participate with 591 providing written informed consent (99%).

#### Assessment of objectively assessed physical activity

Participants were asked to wear the Actigraph GT1M activity monitor (Actigraph, LLC, Pensacola, FL, USA) on the hip during waking times for seven consecutive days. The Actigraph monitor is a uniaxial accelerometer designed to measure change in acceleration in the vertical plane with respect to time. Evenson accelerometry cut-points for adolescents, [14] were chosen to define sedentary time (≤ 100 counts per minute), moderate (≥ 2296 counts per minute) and vigorous physical activity (≥ 4012 counts per minute). A 20-min period of consecutive “zero” counts was used to indicate non-wearing time, [15]. In line with previous research, [16] days with fewer than 10 h of wear time were excluded. Participants with at least one valid day were included. Actilife, version 6.7.0 was used to process data.

#### Assessment of self-reported sedentary behaviours

Interviewer-administered questionnaires were used to measure adolescents’ habitual time spent per day in the following SB: TV viewing, non-active video games (games played sitting), and computer use. Response options were: (1) none (“I do not do this”); (2) 1 to 2 h; (3) 2 to 3 h; or (4) more than 3 h. These questions were adapted from the Arab Teen Lifestyle Study questionnaire [17].

#### Socio-demographic information

Researchers used an interviewer-administered questionnaire to collect participant’s socio-demographic information (date of birth, gender, school grade, governorate and maternal education). Maternal education level was measured using one of the following categories: illiterate, read and write, intermediate, secondary, university or higher education. Maternal education is one of the most commonly used indicators of socioeconomic position in epidemiological studies with adolescents [18].

#### Outcomes

Weight and height were measured in all participants twice and the average was computed. Weight was measured (light clothing, emptied pockets and without shoes), to the nearest 0.1 kg using a SECA (Germany) electronic scale, model 813. Height was measured (without shoes, in bare or socked feet) to the nearest 0.1 cm using a SECA (Germany) portable stadiometer, model 217 whilst standing upright. Blood pressure was measured in the morning on the right upper arm of participants using the appropriate cuff and the Omron HEM 907XL (USA) digital BP monitor (no fasting was required). Prior to the measurements, participants were asked to sit quietly for 3 min with their feet flat. Two readings were then taken with 1 min in between the readings [19].

#### Data handling and statistical analysis

To estimate the time spent in each SB, responses to the questionnaire categories were coded as: none = 0 h/day; 1 to 2 h = 1.5 h/day; 2 to 3 h = 2.5 h/day; more than 3 h = 3 h/day. Age and sex-specific BMI Z-scores were calculated using the WHO AnthroPlus software [20] (WHO 2009). Blood pressure was determined as the mean of the two readings. For maternal education, responses were categorised as: (1) low: included illiterate, read and write level and intermediate level (Grades 7–9); (2) medium: included secondary level (Grades 10–12); (3) high: at least University degree.

Descriptive results are shown as median and inter-quartile range (or mean and standard deviation) for continuous variables and percentages for categorical variables. We tested for interactions between sex and the exposure variables in relation to the study outcomes. As we found significant interactions between TV viewing and sex with BMI, we stratified all analyses by sex. We used multiple linear regression analyses to examine associations between each sedentary exposure (TV viewing, videogames, computer use, total screen-time and objectively measured sedentary time) and outcome (BMI and systolic blood pressure). The models were adjusted for age, sex (in analyses of the total sample), governorate, maternal education, MVPA and accelerometer wear time. For completeness, we also examined the association between MVPA and the two outcomes adjusted for the same confounders as above plus sedentary time. We checked residuals for normality, independence, homoscedasticity and linearity. Two-tailed statistical significance was set at the 5% level. Analysis were performed in SPSS version 24.0’ (IBM Corp, Armonk, NY, USA).

### Results

#### Sample characteristics

From 591 students who provided a written informed consent, 435 students had valid data for both questionnaire-based SB and the outcomes. For objectively measured SB, 84 individuals did not meet the inclusion criteria for accelerometry, leaving 351 adolescents in the final analysis. Descriptive characteristics of the 435 participants, including 201 boys (median age: 15.9 years) and 234 girls (median age: 16.0) are presented in Table 1. A substantial proportion of the sample were overweight or obese: approximately 60% of boys and 48% of girls. Girls reported more time (h/day) TV-watching and using a computer than boys, whereas boys reported more time videogaming. Objectively-measured sedentary time was significantly greater in girls. MVPA was approximately two times greater in boys than girls. Additional descriptive data of questionnaire-based and objectively measured SB time has been reported previously [16].

**Table 1:** Characteristics of participants in the study of health and activity among adolescents in Kuwait (SHAAK)

	Boys (n = 201)	Girls (n = 234)
Age (year)	15.9 (14.7–17.3)	16.0 (14.6–17.6)
Height (cm)	169.0 (168.0–171.0)***	156.0 (155.0–157.0)
Weight (kg)	70.8 (58.9–91.3)***	58.4 (46.1–67.5)
BMI (kg m^−2^)	24.4 (20.6–30.8)**	23.3 (19.1–26.9)
BMI for age Z-score	1.31 ± 1.60***	0.81 ± 1.47
Weight status based on the IOTF		
Overweight/obesity (% total sample)	24.3/35.5	31.8/15.9
Systolic blood pressure (mmHg)	115.6 ± 19.91***	101.5 ± 12.39
Maternal education level (n, %)		
Low^a^	15 (7.5)	25 (10.7)
Medium^b^	27 (13.4)	35 (15.0)
High^c^	159 (79.1)	174 (74.4)
Questionnaire based behaviour		
TV-viewing (h/day)	1.5 (1.5–2.5)	2.5 (1.5–2.6)***
Videogames (h/day)	1.5 (0–1.5)***	0 (0–1.5)
Computer use (h/day)	0 (0–1.5)	1.5 (0–1.5)**
Total screen-time (h/day)	3 (1.5–4.5)	4 (3–5.5)
	**Boys (n** **=** **162)**	**Girls (n** **= 189)**
Accelerometry		
Wear time (h/day)	13.9 (11.9–15.9)	14.3 (12.0–16.6)
Sedentary time (min/day)	492 (431–565)	567 (489–635)***
Moderate to vigorous (min/day)	19 (10.4–30.6)***	8.75 (4.8–16.7)

Continuous variables are shown as median (interquartile range) or mean (standard deviation) according to their distribution. n = number of participants. For categorical variables, n and percentages are shown. Maternal education level: ^a^ Low, read and write, intermediate (Grades 7–9); ^b^ Medium, secondary (Grades 10–12); ^c^ High, at least University degree. Total screen-time = TV viewing + Videogames + Computer use. P-values for sex differences using Mann–Whitney U Test (non-parametric) or Chi Square Test (categorical): *≤ 0.05; **≤ 0.01; ***≤ 0.001

*IOTF* International Obesity Task Force

#### Associations between sedentary behaviours and BMI

In girls, there was no evidence of an association between TV viewing, computer use, total screen-time or objectively measured sedentary time and BMI age-specific Z-score (Table 2).

**Table 2:** Associations between BMI and self-reported or objectively assessed sedentary time or MVPA using linear regression in a sample of Kuwaiti adolescents

	MVPA	Sedentary time	TV viewing	Videogames	Computer use	Total screen-time
*Girls*	n = 234	n = 229	n = 234	n = 234	n = 232	n = 232
β (95% CIs)	0.001 (− 0.014 to 0.017)	− 0.003 (− 0.006 to 0.001)	− 0.17 (− 0.44 to 0.11)	− 0.25 (− 0.48 to − 0.03)	0.10 (− 0.12 to 0.31)	− 0.07 (− 0.20 to 0.05)
P-value	0.857	0.14	0.24	0.03	0.39	0.22
*Boys*	n = 201	n = 190	n = 201	n = 201	n = 199	n = 199
β (95% CIs)	0.001 (− 0.014 to 0.017)	0.03 (0.00 to 0.06)	− 0.03 (− 0.32 to 0.26)	− 0.35 (− 0.58 to − 0.13)	− 0.02 (− 0.29 to 0.24)	− 0.12 (− 0.25 to 0.01)
P-value	0.857	0.04	0.83	0.002	0.85	0.06
*All sample*	n = 435	n = 419	n = 435	n = 435	n = 431	n = 431
β (95% CIs)	− 0.046 (− 0.01 to 0.008)	0.001 (− 0.01 to 0.002)	− 0.13 (− 0.33 to 0.06)	− 0.18 (− 0.42 to − 0.11)	− 0.02 (− 0.19 to 0.14)	− 0.14 (− 0.20 to − 0.028)
P-value	0.56	0.46	0.17	0.01	0.78	0.01

Coefficients represent the change in one unit of BMI age Z-score for an extra 1 h spent per day in TV viewing or videogames or computer use or total screen-time or sedentary time. The model was adjusted for age, sex (in the analysis of the total sample), governorate, maternal education and moderate to vigorous physical activity (MVPA) and accelerometer wearing time. MVPA estimates were derived from the same model, i.e. they were adjusted for age, sex, governorate, maternal education, sedentary time, and accelerometer wearing time. Total screen-time = TV viewing + Videogames + Computer use

*OR* odds ratio, *CI* confidence intervals

In boys, we did not find evidence of an association between TV viewing, computer use or total screen-time and BMI age-specific Z-score. The only exception was a significant, positive association between objectively measured sedentary time and BMI age-specific Z-score in the fully adjusted model (age, governorate, maternal education, MVPA and accelerometer wearing time): β (95% CIs) 0.03 (0.00 to 0.06). For videogames we found evidence of an inverse association with BMI age Z-score in both sexes [girls β (95% CIs) − 0.17 (− 0.48 to − 0.04); boys − 0.24 (− 0.57 to − 0.12)].

#### Associations among sedentary behaviours and blood pressure

In both sexes, there was no evidence of associations between measures of SB (TV viewing, videogames, computer use, total screen-time and sedentary time by accelerometry) and systolic blood pressure (Table 3).

**Table 3:** Associations between systolic blood pressure and self-reported or objectively assessed sedentary time or MVPA using linear regression in a sample of Kuwaiti adolescents

	MVPA	Sedentary time	TV viewing	Videogames	Computer use	Total screen-time
*Girls*	n = 234	n = 229	n = 234	n = 234	n = 232	n = 232
β (95% CIs)	0.09 (− 0.04 to 0.22)	0.02 (− 0.01 to 0.05)	0.03 (− 2.34 to 2.38)	1.57 (− 0.33 to 3.47)	1.47 (− 0.37 to 3.31)	0.91 (− 0.11 to 1.94)
P-value	0.20	0.27	0.98	0.10	0.12	0.08
*Boys*	n = 201	n = 190	n = 201	n = 201	n = 199	n = 199
β (95% CIs)	0.04 (− 0.07 to 0.15)	0.01 (− 0.02 to 0.04)	0.49 (− 2.06 to 3.03)	0.31 (− 1.73 to 2.35)	0.92 (− 1.40 to 3.25)	0.45 (− 0.71 to 1.62)
P-value	0.46	0.59	0.71	0.77	0.43	0.44
*All sample*	n = 235	n = 419	n = 435	n = 435	n = 431	n = 431
β (95% CIs)	0.17 (0.05 to 0.23)	− 0.01 (− 0.02 to 0.01)	− 0.11 (− 2.06 to 3.03)	1.81 (0.30 to 3.33)	0.04 (− 1.5 to 1.64)	0.19 (− 0.66 to 1.04)
P-value	0.002	0.45	0.05	0.02	0.22	0.66

Coefficients represent the change in one mmHg of systolic blood pressure for an extra 1 h spent per day in TV viewing or videogames or computer use or total screen-time or sedentary time. The model was adjusted for age, sex (in the analysis of the total sample), governorate, maternal education and moderate to vigorous physical activity (MVPA). MVPA estimates were derived from the same model, i.e. they were adjusted for age, sex, governorate, maternal education, and sedentary time. Total screen-time = TV viewing + Videogames + Computer use

*OR* odds ratio, *CI* confidence intervals

Finally, we performed sensitivity analyses including both sexes to increase the statistical power of our analyses (and adjusting by age, sex, governorate, maternal education, MVPA and wear time) and there was no difference in the direction of our results (i.e. for total sedentary time and BMI: β (95% CIs) 0.001 (0.00 to 0.003) p = 0.07; or for total sedentary time and BP: β (95% CIs) 0.01 (− 0.001 to 0.025) p = 0.08).

#### Moderate to vigorous physical activity

MVPA was not associated with BMI or blood pressure when boys and girls were analysed separately. When the two sexes were combined, MVPA was associated with systolic blood pressure in the fully adjusted model (age, governorate, maternal education and accelerometer wearing time): β (95% CIs) 0.017 (0.05 to 0.23) p = 0.002).

### Discussion

This study is the first to examine associations between objectively measured SB and cardio-metabolic risk markers in a sample of adolescents from Kuwait. We found limited evidence of an association between objectively measured SB and adiposity or blood pressure. With the exception of adiposity in boys, our findings are consistent with several major accelerometry studies that did not find associations of accelerometry based sedentary time and clusters of cardiometabolic outcomes [21, 22]. Self-reported SB activities (TV viewing and computer use) were also not associated with adiposity risk, mirroring the results of a previous study conducted in Kuwait [23]. In the United Kingdom and United States, TV viewing has been associated with adiposity in children and adolescents, possibly due to exposure to advertisements for unhealthy food and beverage products [7, 8]. Perhaps, TV viewing in Kuwait does not expose adolescents to such food and beverage advertisements, which may explain the difference in our findings.

On the other hand, we found an inverse association between time spent playing videogames and adiposity, where 1 h/day playing videogames was associated with a lower BMI-Z score in girls and boys. This finding contradicts literature from some Western countries, that has reported non-significant associations, [11, 12, 24] or direct associations between videogames and adiposity [8, 10]. As partaking in non-active videogames elicits a very low activity energy expenditure, further research is needed to understand the inverse association with adiposity in Kuwaiti adolescents. It is possible that adolescents who spent more time playing videogames were less likely to eat snacks compared with those who spent more time performing other SB. Finally, the null findings regarding objectively measured SB or self-reported TV viewing and systolic blood pressure are convergent with literature from high-income countries [6, 25].

Our findings indicate that there were limited associations between SB and adiposity and systolic blood pressure in a sample of Kuwaiti adolescents, a findings that is relatively consistent with several large recent observational studies conducted in Western countries.

## Limitations

The study has several important limitations that must be noted. Firstly, our sample only covered three of the six governorates of Kuwait and as such, may not be representative of the broader population. Secondly, the reliability and validity of our SB questionnaire has not been established. However, it remains a challenge to draw robust conclusions about the reliability of self-reported sedentary behaviours due to the use of unstandardized methods in the literature (type and aspect of sedentary behaviour assessed, the period of recall required, the method of administration, the time lapse between assessments and method of analyses) [26]. Maternal education as a surrogate of child SES has not been empirically validated in Kuwait. A standard limitation of all similar studies in the field is that waist-worn accelerometers captures both sedentary behaviour and sitting, what has been termed “stationery behaviour”. Finally, the use of a cross-sectional study design precludes the determination of a causal relationships between sedentary behaviour and adiposity or blood pressure. However, our study provides important preliminary evidence as few studies have been conducted in Middle Eastern countries. Future studies with longitudinal follow-up are warranted.

## Data Availability

The datasets used and/or analysed during the current study are available from the corresponding author on reasonable request.

## Abbreviations

BMI: body mass index
CI: confidence intervals
MVPA: moderate to vigorous physical activity
OR: odd’s ratio
SB: sedentary behaviour
TV: television
